# 119 Addressing inequalities in participation: Developing an inclusive sport and physical activity system across Wales, UK

**DOI:** 10.1093/eurpub/ckae114.128

**Published:** 2024-09-26

**Authors:** Diane Crone, Nick Cavill, Nicola Bolton, Paul Sellars, Tabitha Dickson

**Affiliations:** Cardiff Metropolitan University, Cardiff, United Kingdom; Cavill Associates, Stockport, England, UK; Cardiff Metropolitan University, Cardiff, United Kingdom; Cardiff Metropolitan University, Cardiff, United Kingdom; Cardiff Metropolitan University, Cardiff, United Kingdom

## Abstract

**Purpose:**

Sport and physical activity participation rates remain low and are particularly apparent in areas of deprivation, among girls and women, those with a disability and minority ethnic populations. In collaboration with Sport Wales (the Welsh Government sponsored body for sport in Wales), this project seeks to facilitate the development of an inclusive sport and physical activity system. Its aim is to identify key organisations and potential leaders where participation in sport and physical activity is low and engage them in finding solutions to these challenges. A specific objective of the project is to use insight and engagement from under-represented groups to drive physical activity and sport participation, amongst children and young people, forwards.

**Project description:**

The project, undertaken in two phases, was commissioned by Sport Wales. It commenced in September 2023 and is due to finish in late 2024.

Phase one includes a social network analysis via an online survey to map the existing sport and physical activity network in Wales. Both a formal and informal distribution strategy for the survey was adopted. The survey seeks to ensure sufficient responses to map the network of key stakeholders and identify previously unknown organisations and individuals. The mapping should enhance our understanding of the current network and identify potential new leaders.

Phase two includes the development of a national stakeholder engagement group, made up of the organisations and individuals identified in phase one. This group will act as an advisory group to Sport Wales and produce an engagement plan to inform more effective strategies to target inequalities and under-representation in sport and physical activity participation. A process evaluation is embedded into the project.

**Conclusions and implications:**

Identifying the organisations and leaders in sport and physical activity in areas where participation is low, and actively engaging them in a leadership group reflects a new approach. It demonstrates a commitment by a national organisation to foster collaboration and seek alternative solutions. The project has the potential to be innovative by developing new ways of working and providing solutions to address ongoing and significant challenges in sport and physical activity participation.

